# Challenging Diagnosis of a Patient with Two Novel Variants in the *SYNE1* Gene

**DOI:** 10.3390/ijms251910841

**Published:** 2024-10-09

**Authors:** Anna Kuchina, Aysylu Murtazina, Artem Borovikov, Dmitrii Subbotin, Sergey Bardakov, Maria Akhkiamova, Aleksandra Nikolaeva, Olga Shchagina, Sergey Kutsev

**Affiliations:** 1 Research Centre for Medical Genetics, 115478 Moscow, Russia; murtazina@med-gen.ru (A.M.); borovikov33@gmail.com (A.B.); dmsubbotin@med-gen.ru (D.S.); albmasha@gmail.com (M.A.); nikolaevaepi@gmail.com (A.N.); schagina@dnalab.ru (O.S.); kutsev@mail.ru (S.K.); 2 Department of Neurology, S.M. Kirov Military Medical Academy, 194044 St. Petersburg, Russia; epistaxis@mail.ru

**Keywords:** ataxia, *SYNE1*, hyperCKemia, neuropathy

## Abstract

We report a case of *SYNE1*-associated autosomal recessive spinocerebellar ataxia (SCAR8) presenting with a complex multisystemic phenotype, including highly elevated creatine kinase levels and lower-leg muscle atrophy. In addition to identifying two novel pathogenic variants in the *SYNE1* gene, whole-exome sequencing revealed three variants of uncertain significance in the *DYSF* gene. Electromyography and muscle magnetic resonance imaging indicated a neurogenic pattern of muscle involvement. These findings, along with the segregation analysis of the variants, allowed us to exclude *DYSF*-associated muscular dystrophy; however, we cannot entirely rule out the possibility that the *DYSF* gene variants may act as modifiers of the patient’s phenotype.

## 1. Introduction

Spinocerebellar ataxias (SCAs) comprise a large group of hereditary, heterogeneous, neurodegenerative diseases primarily characterized by ataxia resulting from degenerative changes in the cerebellum. To date, 66 genes have been associated with SCAs, with most cases inherited in an autosomal dominant or recessive manner. The association between the *SYNE1* gene and autosomal recessive spinocerebellar ataxia type 8 (SCAR8) was first identified by Gros-Louis et al. in 2007 [1].

The majority of reported pathogenic variants leading to SCAR8 are loss-of-function (LoF) mutations, primarily nonsense or frameshift variants. Missense variants typically result in disease only when present in a compound heterozygous state with an LoF variant. In addition to ataxia, the *SYNE1* gene is associated with two other phenotypes; biallelic LoF variants are linked to myogenic-type multiple congenital arthrogryposis [2,3,4], and heterozygous missense variants have been reported in patients with Emery–Dreifuss muscular dystrophy [5].

The SCAR8 phenotype can manifest as either a pure ataxic syndrome or as cerebellar ataxia plus, which includes additional symptoms. The classical phenotype of SCAR8 may be modified by a range of extracerebellar symptoms, including upper- and lower-motor neuron involvement, respiratory disorders, and developmental delay, among others. In some instances, these manifestations can contribute to a more severe disease course and, in rare cases, may even lead to sudden cardiac arrest [6,7,8]. Intellectual disabilities are less frequent in patients with SCAR8 [6]. Several cases with mildly elevated creatine kinase (CK) levels have also been described [7,8].

Dysferlinopathy encompasses a broad spectrum of autosomal recessive muscle disorders. The phenotypic manifestations of dysferlinopathies range from isolated hyperCKemia to muscular dystrophy with varying patterns of muscle involvement [9]. The *DYSF* gene is primarily associated with either limb-girdle muscular dystrophy type R2 or Miyoshi distal myopathy, both of which are characterized by significantly elevated CK levels.

In this report, we present the clinical manifestations and genetic findings of a patient with two novel compound heterozygous LoF variants in the *SYNE1* gene. Additionally, three missense variants in the *DYSF* gene were identified, which may contribute to some of the patient’s symptoms.

## 2. Case Report

The proband is a 15-year-old male who presented with complaints of difficulty maintaining balance while walking, severe coordination impairment, hand tremors, significant muscle wasting in the hands, and impaired handwriting. He was born to a Russian family and has a healthy older brother. The parents are non-consanguineous. The patient was born at 39 weeks of gestation from the third pregnancy, which was complicated by a threatened miscarriage during the second trimester. His birth weight was 3500 g, and his length was 50 cm, with an Apgar score of 8/9.

Motor development was delayed. He began rolling over from his back to his abdomen at 8 months, sitting at 9 months, and walking at 14 months. The parents also noted a weak grip in infancy, and atrophy of the hand muscles developed in early childhood. Mental and speech development were consistent with age-appropriate milestones. At age 10, he began experiencing difficulty maintaining balance while riding a bicycle or scooter, followed by the onset of gait disturbances and hand tremors. The patient has been monitored by an ophthalmologist since childhood for partial cataracts in both eyes. No abnormalities were detected in the cardiovascular or respiratory systems.

**Figure 1:**
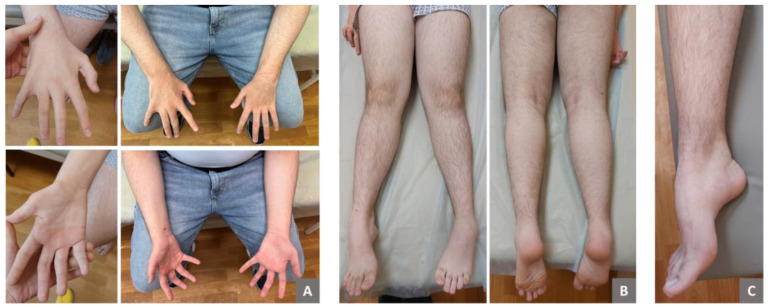
Clinical presentations of a patient with *SYNE1*-associated spinocerebellar ataxia. The images show distal muscle atrophy in the upper and lower limbs (**A**–**С**). Notable features include atrophy of the thenar and interosseous muscles of the hands, partial cutaneous syndactyly of the 4th and 5th fingers on the right hand, bilateral cone-shaped fingers, and medial deviation of the thumbs (**A**). Additionally, asymmetric atrophy and valgus deformity of the lower legs (**B**), as well as pes cavus (**C**), are evident.

At the age of 15, the patient exhibited several phenotypic features, including a low hairline on the forehead, synophrys, and hypoplasia of the midface. Apart from mild dysarthria, there were no signs of cranial nerve involvement. Examination revealed atrophy of the distal parts of the forearms and hands (Figure 1), as well as asymmetric atrophy and hypotonia of the gastrocnemius muscles (more pronounced on the left side; Figure 1). Weakness of both finger extensors and flexors was noted, with muscle strength rates as 3/5 on the Medical Research Council scale. Arm and leg deep-tendon reflexes were hyperactive, and pathological reflexes were present. Generalized hyperesthesia was also observed, although proprioception remained intact. The patient demonstrated postural tremors and truncal and limb ataxia and was unable to perform a tandem walk. Additionally, he exhibited pes cavus and ankle-tendon retraction on both sides. No muscle fasciculations were observed.

CK levels were repeatedly elevated, ranging from 1600 to 2300 U/L (normal range: 40-200 U/L). Nerve conduction studies and needle EMG demonstrated isolated axonal involvement of motor nerves, with a distinctly neurogenic pattern primarily affecting the upper limb muscles. Pathological spontaneous activity was moderate, including fibrillation potentials and complex repetitive discharges. Electrocardiography and echocardiography showed no abnormalities, and pulmonary function tests were normal. Ophthalmological examination revealed simple hyperopic astigmatism and partial cataracts in both eyes.

**Figure 2:**
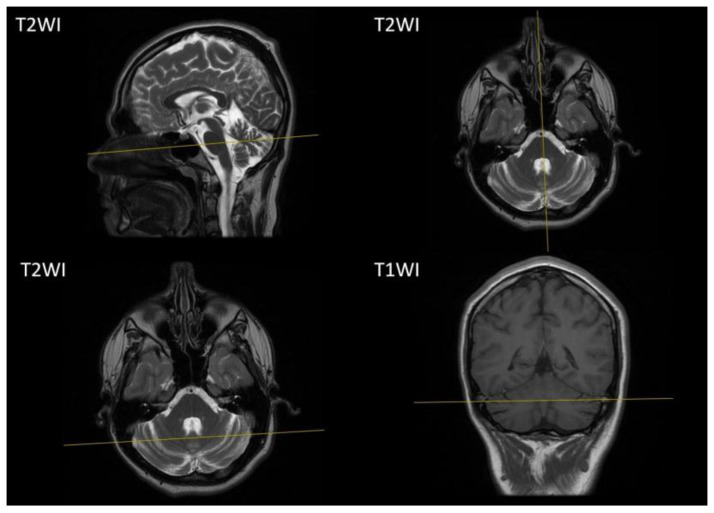
Brain MRI of our proband. Sagittal, axial T1-weighted images and coronal T2-weighted images demonstrate atrophic changes of the cerebellum. T1WI, T1-weighted image; T2WI T2-weighted image.

A brain MRI at the age of 14 years showed atrophic changes in the cerebellum (Figure 2), while other brain structures and the spinal cord appeared unremarkable.

**Figure 3:**
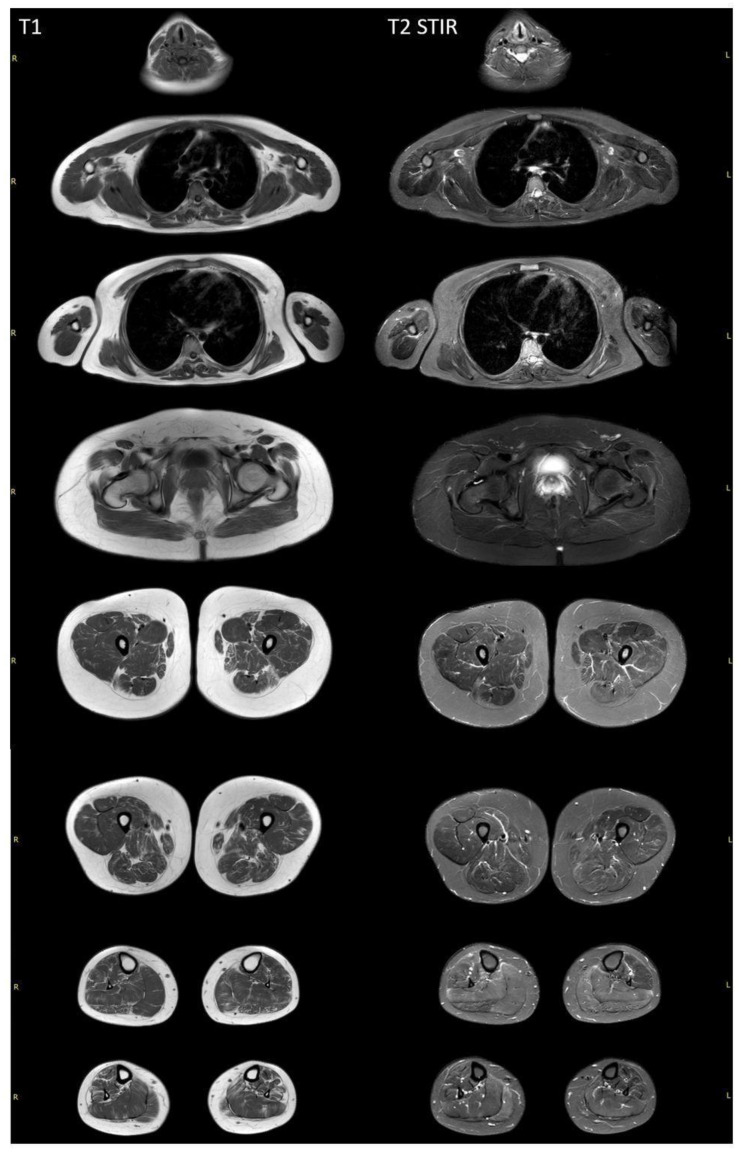
Whole-body muscle MRI of a patient with *SYNE1*-associated spinocerebellar ataxia. T1-weighted MRI revealed diffuse fatty replacement in lower limb muscles, suggesting a neurogenic pattern. In all lower limb muscles, a moth-eaten appearance is visualized, with the exception of the adductor longus and gluteus major bilaterally. Vasti muscles, as well as the medial heads of the right gastrocnemius and soleus muscles, are more preserved. The T2 STIR-weighted images demonstrate a hyperintense signal in the posterior compartment of the lower leg muscles. There are no signs of the involvement of neck, trunk, or upper limb muscles.

Whole-body muscle MRI revealed a diffuse pattern of fatty replacement in the lower limb muscles on T1-weighted images (Figure 3), indicative of a neurogenic pattern. The relatively spared muscles include the adductor longus, gluteus maximus, vasti muscles, medial head of the right gastrocnemius, and soleus muscles. T2 STIR-weighted images demonstrated a hyperintense signal in the posterior compartment of the lower leg muscles, suggestive of neurogenic muscle edema. No involvement was observed in the neck, trunk, or upper limb muscles.

Given the presence of various clinical manifestations, including signs of myopathy, such as muscle wasting and highly elevated CK levels, as well as ataxic syndrome, the possibility of multiple hereditary diseases was considered. The proband underwent whole-exome sequencing (WES) as the initial stage of genetic testing.

As a result, two heterozygous LoF variants were identified in the *SYNE1* gene (NM_182961.3), namely c.24979dup (p.Asp8327GlyfsTer18) and c.19468C>T (p.Gln6490Ter). Additionally, three heterozygous variants were detected in the DYSF gene (NM_001130987.2), namely c.386G>A (p.Gly129Glu), c.4253C>G (p.Pro1418Arg), and c.5143G>T (p.Ala1715Ser). The p.Gly129Glu variant has been previously reported and is present at a higher-than-expected frequency in the gnomAD dataset. However, it has been associated with dysferlinopathy in several studies. The other two missense variants in the *DYSF* gene are novel. We decided to proceed with family analysis for all five of these variants to exclude the possibility of all three variants occupying the cis position.

When performing segregation analysis using Sanger sequencing, it was revealed that variant c.386G>A in the *DYSF* gene was inherited from the mother and that variants c.4253C>G and c.5143G>T were inherited from the father, in addition to being identified in the healthy brother. The variants in the *SYNE1* gene were in a compound heterozygous state.

## 3. Discussion

Most of the reported variants associated with the SCAR8 are biallelic loss-of-function (LoF) variants distributed throughout the SYNE1 gene. In our patient, both novel variants were also found to be LoF variants and likely to lead to an absence of the protein due to nonsense-mediated decay (NMD) mechanisms. Supporting this, a study by M. Synofzik et al. demonstrated that SYNE1 did not escape NMD. In their work, they confirmed the degradation of SYNE1 mRNA via NMD in a patient with biallelic truncating variants (p.G4752Efs10 and p.L132), reinforcing the role of NMD in the elimination of SYNE1 transcripts with truncating variants [6].

As in other reported cases, the primary clinical feature in our patient was ataxia, which manifested at the age of 10 years [6]. Initially, based on the proband’s phenotype and WES results, we could not definitively exclude the possibility of a double-trouble condition involving a combination of two hereditary diseases, namely SCAR8 and dysferlinopathy. With the exception of the elevated CK levels, which reached 2300 U/L, the patient’s phenotype aligns with SCAR8, presenting with truncal and limb ataxia, atrophy and weakness in the distal parts of the arms, asymmetric atrophy of the gastrocnemius muscles, pyramidal signs, sensory disturbances such as hyperesthesia, postural tremor, foot deformity, and facial dysmorphism.

To the best of our knowledge, elevated CK levels are not typically associated with SCAR8. Although elevated CK levels have been reported in patients with biallelic variants in the SYNE1 gene, these levels are generally lower than those observed in our proband. For example, a clinical report of several Chinese patients with SYNE1 variants and elevated CK levels documented a maximum CK value of 755.7 U/L [7].

Previously, it has been suggested that variants in the *DYSF* gene could be responsible for isolated hyperCKemia [9,10,11]. Following segregation analysis in our case, we determined that heterozygous variant p.Gly129Glu, with a relatively high allele frequency, was in a trans position with two other heterozygous variants, namely p.Pro1418Arg and p.Ala1715Ser. Variant p.Gly129Glu was identified in the gnomAD v.2.1.1 database in a homozygous state in 21 carriers. This variant had previously been reported in patients with limb-girdle muscular dystrophy and hyperCKemia [12,13]. However, it was later reclassified by other research groups as benign, primarily due to its allele frequency in population databases [14]. Among the variants found in the cis position, the p.Pro1418Arg variant exhibited a higher score in in silico predictions. Its predicted probability of being damaged was moderate, with a REVEL score of 0.72, an AlphaMissense score of 0.8542, and a CADD score of 24.

Muscle MRI and needle EMG did not support a diagnosis of dysferlinopathy. Specifically, MRI did not reveal the characteristic pattern of muscle involvement typically associated with dysferlinopathy. Instead, MRI of the leg muscles displayed diffuse changes consistent with neurogenic muscle damage. EMG did not show myogenic changes, and the findings were consistent with those reported in the literature for SCAR8.

The elevation of CK levels to 2300 U/L in our patient may suggest pronounced axonal neuropathy. However, considering previous work indicating that hypomorphic variants in the DYSF gene could be linked to hyperCKemia, it is also important to consider that DYSF variants may influence the patient’s phenotype [9,10,11,15,16,17,18]. DYSF plays a crucial role in cytoskeletal muscle remodeling, while SYNE1 is a key component of the LINC complex, which facilitates communication between the nucleus and the cytoskeleton [18,19]. Given these roles, DYSF variants might act as modifying factors that impact the clinical presentation. Therefore, further functional studies are essential to explore this possibility and validate the influence of DYSF variants on the phenotype.

## 4. Materials and Methods

Our article presents the clinical and genetic characteristics of a 15-year-old male patient from a non-consanguineous Russian family diagnosed with *SYNE1*-associated autosomal recessive spinocerebellar ataxia. The diagnostic workup included laboratory tests such as creatine kinase (CK) level measurement, nerve conduction studies, needle electromyography (EMG), and whole-body muscle magnetic resonance imaging (MRI). DNA diagnostics were conducted using whole-exome sequencing, with all findings confirmed by Sanger sequencing. All biological samples have been deposited in the Moscow Branch of the Biobank “All-Russian Collection of Biological Samples of Hereditary Diseases”.

## 5. Conclusions

We presented a case involving two novel LoF variants in the *SYNE1* gene, leading to a diagnosis of SCAR8 after thorough examination of the proband. However, elevated CK levels in our proband were the only finding that could potentially be associated with *DYSF* variants or could represent an expansion of the phenotypic spectrum of SCAR8.

## Data Availability

The datasets for this article are not publicly available due to concerns regarding participant/patient anonymity. Requests to access the datasets should be directed to the corresponding author.
